# A dataset of simulated patient-physician medical interviews with a focus on respiratory cases

**DOI:** 10.1038/s41597-022-01423-1

**Published:** 2022-06-16

**Authors:** Faiha Fareez, Tishya Parikh, Christopher Wavell, Saba Shahab, Meghan Chevalier, Scott Good, Isabella De Blasi, Rafik Rhouma, Christopher McMahon, Jean-Paul Lam, Thomas Lo, Christopher W. Smith

**Affiliations:** 1 Western University, London, N6A 3K7 Canada; 2 Goodlabs Studio, Toronto, M5H 3E5 Canada; 3 Department of Economics, University of Waterloo, Waterloo, N2L 3G1 Canada; 4 Polytechique Montreal, Montreal, H3T 1J4 Canada

**Keywords:** Health care, Medical research

## Abstract

Artificial Intelligence (AI) is playing a major role in medical education, diagnosis, and outbreak detection through Natural Language Processing (NLP), machine learning models and deep learning tools. However, in order to train AI to facilitate these medical fields, well-documented and accurate medical conversations are needed. The dataset presented covers a series of medical conversations in the format of Objective Structured Clinical Examinations (OSCE), with a focus on respiratory cases in audio format and corresponding text documents. These cases were simulated, recorded, transcribed, and manually corrected with the underlying aim of providing a comprehensive set of medical conversation data to the academic and industry community. Potential applications include speech recognition detection for speech-to-text errors, training NLP models to extract symptoms, detecting diseases, or for educational purposes, including training an avatar to converse with healthcare professional students as a standardized patient during clinical examinations. The application opportunities for the presented dataset are vast, given that this calibre of data is difficult to access and costly to develop.

## Abstract (summary)

**Table:** 

Measurement(s)	conversations
Technology Type(s)	audio recording and transcription
Factor Type(s)	N/A
Sample Characteristic - Organism	simulated medical exams
Sample Characteristic - Environment	simulation
Sample Characteristic - Location	simulation

## Background & Summary

Artificial Intelligence (AI), including Natural Language Processing (NLP), Machine Learning (ML) models and deep learning tools, are playing an increasingly important role in medicine such as in education, diagnosis and disease classification. However, in order to train NLP models, robust and accurately documented medical conversations are needed. The presented medical conversation data is challenging to obtain, especially in the format of audio files with corresponding processed and transcribed text documents. This dataset can be utilized to benefit the greater community, including academia and the medical industry.

**Fig. 1:**
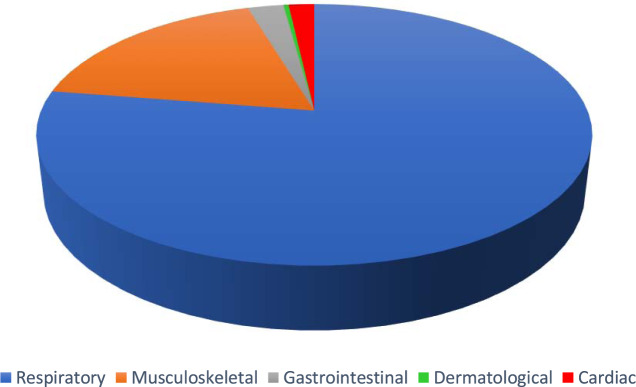
Pie chart demonstrating the proportion of cases in the following categories: respiratory (78.7%, blue), musculoskeletal (16.9%, orange), gastrointestinal (2.2%, grey), cardiac (1.8%, red) and dermatological (0.4%, green).

A team of resident doctors in internal medicine, physiatry, anatomical pathology and family medicine, and senior Canadian medical students created this dataset. The medical interviews were recorded in the format of Objective Structured Clinical Examinations (OSCE)^1^. 272 cases were simulated between the physician and the patient. These cases were recorded and classified into the categories of respiratory, musculoskeletal, cardiac, dermatological, and gastrointestinal diseases. However, the majority of simulations were respiratory cases. Please see Fig. 1 for a visual representation of the types of cases included. These audio recordings were then transcribed, manually corrected for speech to text errors, and an identifier was added to specify the speaker.

Each component of the presented dataset can be used for various purposes. The audio recordings can be used to test the accuracy of transcription tools, and to detect speech-to-text errors. The manually corrected transcripts can be annotated with desired tags to build Named-Entity Recognition (NER) tools in order to train various NLP models. For example, the data can be used to train an NLP model to use avatars instead of the traditional standardized patient to converse with medical students for OSCEs. This has been explored by a study that investigated obtaining word embeddings from an NLP model trained on medical documents and a convolutional neural network (CNN) trained on Question-Answer (QA) systems^2^. However, their models only resulted in an accuracy of 81% in answer selection^2^. The presented dataset may help to increase the accuracy of such an educational model due to the nature of OSCE-simulated medical conversations, the rationale for chosen cases, and manual correction of speech-to-text errors and speaker identification.

A brief literature search demonstrated that Speech Recognition (SR) software studies in the past had shown error rates ranging from 7.4 to 65%^3,4^. However, SR is still necessary to reduce turnaround times and cost-effective reporting of patient-physician interviews^5,6^.

One study stated that recordings made in a controlled environment with speakers simulating a medical conversation while sitting directly in front of a microphone are best for high-quality audio^7^. However, even in these ideal conditions, using conversational speech to train SR software leads to errors due to speech that is not well-formed, disfluencies like false starts, extraneous information, pauses, repetitions, and interruptions^8^. It was also found that SR software trained with medical dictations leads to higher error rates compared to those trained with medical conversations because of the lack of punctuation and grammatical differences in spoken and written language^9,10^. In addition, the transcript produced lacks clear structure because of the natural flow of the conversation^11^ so the transition from one speaker to the next may not be clear^12,13^. To help improve the accuracy of NLP models, the presented dataset countered these issues by producing high-quality audio, minimizing disfluencies, simulating medical conversations through the tested and tried OSCEs and identifying each speaker in the transcripts.

Lastly and most importantly, getting access to medical conversations is a major roadblock for many studies because of the confidential nature of the data^14,15^, government regulations limiting the sharing of data in research, and the issue of data being monetized^16^. Research has been done using large volumes of medical conversations^17,18^, but they are private and not shared due to industrial and research advantages since these datasets are costly to develop^19^. One of the few publicly available large-scale medical dialogue datasets is MedDialog which contains both a Chinese dataset with 3.4 million conversations and an English dataset with 0.26 million conversations covering 96 specialties^20^. The purpose of this dataset was to create medical dialogue systems to assist in telemedicine/online medical forums^20^. While this dataset is open to the public with a large volume, the data is in text format only, does not have a structured approach such as the OSCE and only some conversations conclude with a diagnosis which may have implications in training NLP models for the purposes discussed previously^20^. Additionally, these transcripts are predominately from online medical forums, and do not accurately represent live conversations. The Bristol Archive Project also created a dataset of 327 video-recorded primary care consultations and coded transcripts known as the “One in a million primary care consultations archive” for future research and teaching purposes^16^. This data can be accessed by researchers with ethics approval to develop medical and research training^16^. This dataset is similar to the presented dataset in terms of methodology and content and therefore, can likely be used in combination to increase the accuracy of NLP models^16^. However, this dataset was created exclusively based on the patient population of West England, therefore having implications for generalizability^16^. In summary, robust and accurate medical conversations are of utmost value, and the presented dataset can be a valuable asset to many in academia and the industry.

## Methods

The methodology of developing this dataset can be broken down into the following components:

A) Recording of Simulated Medical Conversations
B) Cleaning of Audio
C) Manual Correction of Transcripts
D) Quality Control

A. Recording of Simulated Medical Conversations

A team of resident doctors in internal medicine, physiatry, anatomical pathology and family medicine, and senior Canadian medical students recorded simulated medical conversations in the format of Objective Structured Clinical Examinations (OSCE) on Microsoft Teams. Unlike traditional clinical exams, the OSCE is a practical and objective approach in the diagnosis and communication of medical conditions, and has the ability to handle unpredictable patient behaviour and seemingly unrelated symptoms^21^. It is often used as a standardized method to test students’ clinical skills.

Cases were divided into the following categories:

- Respiratory cases (designated “RES”)
- Musculoskeletal cases (designated “MSK”)
- Cardiac cases (designated “CAR”)
- Dermatological case (designated “DER”)
- Gastrointestinal cases (designated “GAS”)

**Fig. 2:**
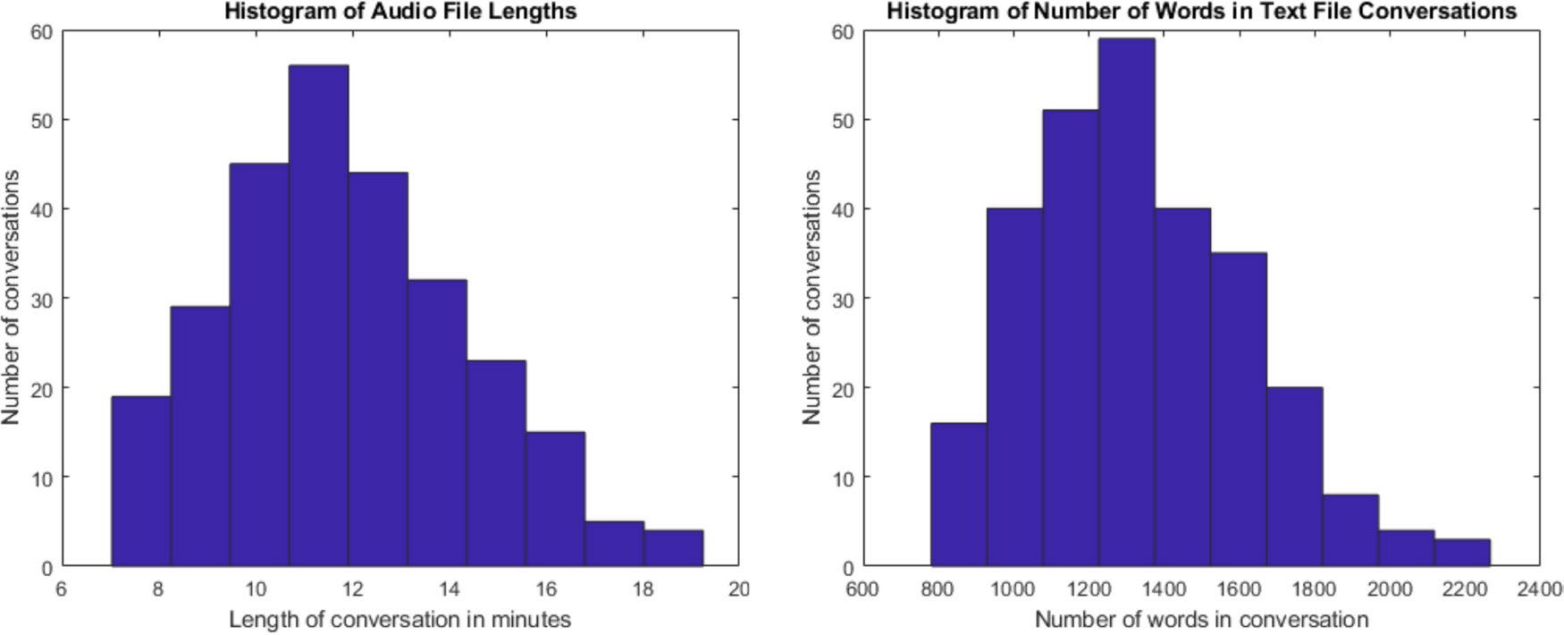
Histograms displaying the number of conversations with their corresponding length of time in minutes (left) and number of words per conversation (right).

272 cases were simulated and recorded (please refer to Fig. 1). The focus of the dataset was respiratory cases (214 cases). In addition, 46 musculoskeletal cases, 5 cardiac cases, 6 gastrointestinal cases and 1 dermatology case were also simulated. Of the total simulated recordings, 57% of the cases involved a male physician and 43% involved a female physician. From the patient perspective, 55% of the simulated cases involved a male patient and 45% involved a female patient. The average duration of each conversation was 11 minutes and 56 seconds. For further details, please refer to Fig. 2 for a histogram of the number of cases corresponding to various lengths of time. The focus was on respiratory cases because most pandemics, including the COVID19 pandemic, are caused by droplet or airborne based respiratory diseases. Therefore, it is crucial to differentiate between a benign cause of malaise such as the common cold from a highly infectious and fatal cause such as COVID19 or Tuberculosis.

In deciding which medical conditions to simulate, two considerations were taken into account; the first being prevalence of the condition, and the second being mortality rate of the condition if left untreated. For example, in simulating respiratory conditions, a common infectious condition is the common cold, most often caused by rhinovirus^22^, whereas a fatal condition if left untreated is a pulmonary embolism^23^. The rationale for these considerations was that physicians are taught to recognize and treat common conditions and to not miss fatal conditions. However, some conditions that are not common or highly fatal were also included within the dataset to represent the diversity of cases seen in the clinic and hospital setting. In addition, COVID19 cases were included to reflect the landscape of current burden of disease in medicine.

Each case was simulated between the acting physician and the acting patient, both being senior medical students or resident doctors. The patient chose a case using the two considerations discussed previously to guide his/her decision, and answered questions posed by the physician. Medical students and resident doctors are not typically assessed on their competence at being a standardized patient. However, they have observed many trained standardized patients during assessed OSCEs and have a good perception of how patients respond in hospital/clinical settings, and they were prompted to answer questions posed by the physician as how patients would respond in a clinical/hospital setting ie. vague responses to open-ended questions and specific responses to direct questions. In addition, they were given the liberty of choosing the age and gender that they wanted to portray keeping in mind the demographic population that would normally present with the condition that they have chosen to portray.

The acting physician was told to take a history as they normally would in the hospital or clinic setting to help inform a differential diagnosis. While it was acknowledged that senior medical students and resident doctors will have slightly different competency levels, they were told to ask baseline questions including symptoms experienced, time of onset, location, severity, quality, associated symptoms, review of systems, past medical history, medication, family history, sexual history and social history including travel, sick contacts, employment, housing, alcohol consumption and recreational drug use. The physician was blinded to the final diagnosis to simulate the clinic and hospital setting and to avoid asking leading questions. Each case was concluded by the physician using information gathered on history taking in order to formulate a differential diagnosis and management plan. It is important to note that although these medical conversations were recorded in the format of OSCEs, the pressures of assessment and evaluation were not a component of these conversations.

B. Cleaning of Audio
  The recorded medical conversations were uploaded to Audacity 3.0.2 (www.audacityteam.org), an open-source audio editing platform, to trim extraneous information, including patient/physician identifiers and any part of the conversation that was not organic. For example, case presentations in which the physician summarized patient age, gender and history of presenting complaints during which he/she was not directly speaking to the patient was trimmed out.
C. Manual Correction of Transcripts
  The recorded medical conversations were uploaded to the “Microsoft Stream” platform for transcription. These transcripts were then manually corrected for speech-to-text errors, including spelling mistakes, grammar mistakes, and incorrect punctuation. For example, a common error picked up in respiratory cases was the term “cough” which was often transcribed as “cost”. Key pieces of information were also added if missed during the speech-to-text transcription phase. For example, the speech-to-text software blacked out the term “sexual” when the physician inquired about sexual health and sexually transmitted infections. Therefore, this was added back to the transcript for completeness. In addition, the text file was manually reviewed to separate physician lines indicated by “D” for doctor and patient lines indicated by “P” in order to delineate the transition between speakers. Live editing occurred while simultaneously listening to the audio files to minimize errors. Table 1 demonstrates an example of part of a transcribed audio recording that was manually corrected.

**Table 1:** An example of part of a transcribed audio recording and manual correction (from RES0051).

Speech to text original transcript	Would you mind starting with
	telling me what brought you in?
	Sure I I have had this cough for
	the past five days and it doesn’t
	seem to be getting any better so.
	I’m I’m just here too.
	Ask you what,
	what it what it possibly could be.
	At has the cough been getting any better?
	Staying the same or getting
	worse over these last five days?
	I think it’s getting worse
Manual Correction	D: Would you mind starting with telling me what brought you in?
	P: Sure, I have had this cough for the past five days and it doesn’t seem to be getting any better so I’m just here to ask you what it what it possibly could be.
	D: Has the cough been getting any better, staying the same or getting worse over these last five days?
	P: I think it’s getting worse.
D. Quality Control

Once the audio was cleaned and transcripts manually corrected by the initial reviewer, a team of two people reviewed the audio files and transcripts in order to ensure that the mistakes discussed in part b and c were not present. This was performed by simultaneously listening to the corresponding audio file while editing the transcript. The American version of English was used for the transcripts.

## Data Records

The simulated medical conversation dataset is available on figshare.com^24^. The dataset is divided into two sets of files: audio files of the simulated conversations in mp3 format, and the transcripts of the audio files as text files. There are 272 mp3 audio files and 272 corresponding transcript text files. Each file is titled with three characters and four digits. RES stands for respiratory, GAS represents gastrointestinal, CAR is cardiovascular, MSK is musculoskeletal, DER is dermatological, and the four following digits represent the case number of the respective disease category.

## Technical Validation

Using the Objective Structured Clinical Examination (OSCE) format for medical conversations facilitated objectivity, consistency, and organization. Medical conversations between resident doctors and medical students followed an overall format of elucidating the following pertinent information: symptoms and respective qualifiers (such as time of onset, location, severity, etc.), associated symptoms, review of systems, past medical history, medications, family history, social history, and other risk factors. During the manual correction of the transcript phase, key pieces of information were added if missed during the speech to text transcription phase, and corrected for spelling errors, grammar mistakes, and other inconsistencies. Speaker transition was also denoted. The audio and transcripts were again reviewed by exhaustively listening to all audio files while manually correcting each transcript after the initial processing of transcript to ensure the text accurately reflected what was said in the audio file. As discussed in Methods, the “physician” was blinded to the final diagnosis in order to simulate the clinic and hospital setting, and to avoid asking leading questions.

## Usage Notes

The presented dataset can be utilized in many ways. The audio recordings can be used to test the accuracy and precision of transcription tools and speech recognition software. By extension, it can be used to detect and fix speech-to-text errors. The manually corrected transcripts can be annotated with desired tags to develop tools such as Named-Entity Recognition (NER) and train NLP models to build educational models. For example, it can be used to train an NLP model to use avatars to converse with medical students or other healthcare professional students for OSCEs by replacing the traditional standardized patient which can have cost and access implications for students and institutions. Overall, this comprehensive dataset can also be used to create an end-to-end system from symptom extraction to disease classification.

High-quality audio of medical conversations is difficult to simulate due to factors such as environment control and microphone position^7^. In addition, high-quality transcripts of medical conversations are difficult to access due to speech-to-text errors of SR software, including spelling errors, grammar mistakes, and disfluencies like false starts, extraneous information, pauses, repetitions and interruptions^8^. The transcribed file also often fails to indicate the transition between speakers^12^. In creating this dataset, special attention was given to all of these drawbacks in order to create a comprehensive dataset that is robust, accurate, easy to understand and applicable to train any NLP model. Most importantly, access to this calibre of data is a major challenge for many researchers because of the confidential nature of the data^14,15^, government regulations that limit data sharing in research, and the issue of data being monetized^16^. Therefore, the presented dataset of comprehensive medical conversations in audio and text formats is a valuable asset to academia and the medical industry.

While there are many benefits to this dataset, as aforementioned, there are limitations to using this data set to train NLP models. The first limitation is the small number of conversations of non-respiratory illnesses. It is important to note that although these medical conversations were recorded in the format of OSCEs, the pressures of assessment and evaluation were not a component of these conversations. This may have implications specifically if these conversations were to be used to train an NLP model to use avatars to converse with medical students or other healthcare professionals for OSCEs. However, as discussed in the methods section, the physician was instructed to ask questions as they would in the hospital or clinic setting and prompted to cover baseline topics as previously discussed. In addition, not having the pressures of a formal evaluation may serve as a benefit in simulating medical conversations as it could allow for more realistic dialogue encountered in the clinic/hospital setting. The patient was given the liberty to choose the age and gender that he/she wanted to portray based on the demographic population that would typically present with his/her chosen condition. This resulted in audio files of the medical student/resident doctor (who were in their twenties) with a voice that does not match an elderly patient if they have chosen to represent that population. This may have implications for its potential use in speech recognition detection for speech-to-text errors as the voice of an elderly patient may be different sounding than a younger patient and thus, may affect the ability/quality of the speech to text function. However, since the audio files are also converted into corrected manuscripts, this should not have any implications for training NLP models to extract symptoms, detect diseases, or for educational purposes, including training an avatar to converse with healthcare professional students as a standardized patient during clinical examinations. In addition, although the OSCE- styled medical conversations are superior to traditional clinical exams in terms of objectivity, precision, and ability to handle unpredictable patient behavior and seemingly unrelated symptoms, they are limited in their ability to simulate real-world patient-physician conversations, which are more complex due to subtle body language, facial cues and other non-verbal presentations. Thirdly, these medical conversations only covered the history-taking part of simulated medical visits. Physical exams were not included in the medical conversation and therefore, there may be limitations in informing a clinical differential diagnosis and management plan. This dataset has 3309 minutes of audio and 272 transcribed texts. Training AI models is data-intensive requiring large amounts of data^25–27^. Therefore, this dataset can be combined with other datasets for the purposes described previously. The user will have to take into consideration transferability and generalizability when combining such data. Lastly, this dataset focussed predominantly on respiratory cases so it does limit usage. However, as discussed previously, the team believed this topic was most relevant given the current burden of disease, particularly the COVID19 pandemic.

## Data Availability

Not applicable to this dataset.
